# Reverse Genetic Screen for Deleterious Recessive Variants in the Local Simmental Cattle Population of Switzerland

**DOI:** 10.3390/ani11123535

**Published:** 2021-12-12

**Authors:** Irene M. Häfliger, Franz R. Seefried, Cord Drögemüller

**Affiliations:** 1 Institute of Genetics, Vetsuisse Faculty, University of Bern, 3012 Bern, Switzerland; irene.haefliger@vetsuisse.unibe.ch; 2 Qualitas AG, Chamerstrasse 56, 6300 Zug, Switzerland; franz.seefried@qualitasag.ch

**Keywords:** cattle, *Bos taurus*, reproduction, breeding, fertility, embryonic lethality, loss-of-function variants, whole-genome sequencing, SNP genotyping

## Abstract

**Simple Summary:**

Today’s Swiss Simmental represents a local dual-purpose breed of cattle. Within closed populations, deleterious variants can reach problematic frequencies, explaining substantial proportions of inbreeding depression. Depletions in homozygous genotypes for certain haplotypes among large cohorts of animals genotyped for the purpose of genomic selection is a widely used approach to pinpoint undesired recessive alleles. In the course of a reverse genetic screen, we aimed to identify single recessive Mendelian variants that potentially affect fertility and rearing success without any phenotypic information available. We detected eleven genome regions showing obvious depletion of homozygosity based on genome-wide SNP data. Furthermore, after performing whole-genome sequencing of selected animals, we propose three candidate causative variants affecting different genes with possibly detrimental effects for embryonic development. The established haplotypes, as well as the identified protein-changing variants, can be directly implemented into breeding practice to avoid the risk of mating carriers and thereby increase breeding success.

**Abstract:**

We herein report the result of a large-scale reverse genetic screen in the Swiss Simmental population, a local dual-purpose cattle breed. We aimed to detect possible recessively inherited variants affecting protein-coding genes, as such deleterious variants can impair fertility and rearing success significantly. We used 115,000 phased SNP data of almost 10 thousand cattle with pedigree data. This revealed evidence for 11 genomic regions of 1.17 Mb on average, with haplotypes (SH1 to SH11) showing a significant depletion in homozygosity and an allele frequency between 3.2 and 10.6%. For the proposed haplotypes, it was unfortunately not possible to evaluate associations with fertility traits as no corresponding data were available. For each haplotype region, possible candidate genes were listed based on their known function in development and disease. Subsequent mining of single-nucleotide variants and short indels in the genomes of 23 sequenced haplotype carriers allowed us to identify three perfectly linked candidate causative protein-changing variants: a SH5-related *DIS3:*p.Ile678fs loss-of-function variant, a SH8-related *CYP2B6:*p.Ile313Asn missense variant, and a SH9-related *NUBPL:*p.Ser143Tyr missense variant. None of these variants occurred in homozygous state in any of more than 5200 sequenced cattle of various breeds. Selection against these alleles in order to reduce reproductive failure and animal loss is recommended.

## 1. Introduction

Simmental is a globally recognized cattle breed, originating in the Simmental valley in the canton Bern in Switzerland. Autochthonous of Switzerland, today up to 40 million cattle worldwide are designated as members of the Simmental breed. However, Simmental are characterized by very different local breeding objectives (http://wsff.info; accessed on 24 October 2021). In the 19th century, crossbreeding of local cattle with Simmental cattle exported from Switzerland led to Fleckvieh populations in neighboring countries, including Austrian and German Fleckvieh, French Montbeliarde, and Italian Pezzata Rossa cattle. In the 20th century, introduction of animals of the Holstein breed into the Swiss Simmental population led to today’s Fleckvieh population in Switzerland (Swiss Fleckvieh). A recent analysis of the population structure of Swiss cattle showed a clear differentiation between today’s Swiss Simmental cattle and all other Swiss cattle populations, including the modern Swiss Fleckvieh [1,2]. Various historically younger Central European Simmental populations that descended from the Swiss Simmental can also be clearly distinguished from today’s Swiss Simmental animals, which showed the highest inbreeding level [3]. In contrast, recent studies found a comparatively low degree of genomic inbreeding in purebred Swiss Simmental that might be explained by the continued use of natural service sires, which is likely the major reason for their remarkably high level of genetic diversity although this population has been closed for a long time [1,2]. Therefore, the current so-called Original Simmental breed of Switzerland represents a unique purebred population. At the end of 2020, more than 23 thousand dual-purpose animals in the Simmental population were registered in the Swiss herdbook (https://www.swissherdbook.ch/fileadmin/Domain1/PDF_Dokumente/05-Statistiken-Formulare/53-Jahresstatistik/1_Wichtigste_Zahlen/D_sh_JS_2020_HBZahlen_web.pdf; accessed on 24 October 2021).

A universal problem in cattle breeding is reproductive failure. It was shown that the reproduction success is negatively associated with production traits [4,5]. These effects have been thoroughly studied and possible reasons are selection programs focusing on production traits coupled with negative correlation with reproductive traits [6]. Regarding early pregnancy loss in Simmental cattle, e.g., a Croatian study described the body condition score, parity, and milk yield as important influencing factors [7]. Lower reproductive success is an economic problem for farmers and a major reason for cattle slaughter [8,9]. To tackle fertility issues, one approach is to apply a so-called reverse genetic screen, where only genomic data is used rather than phenotypic information. A whole-genome sequencing (WGS)-based approach was suggested by Charlier et al. (2016), where sequencing data of entire genomes is mined for variants affecting fertility by causing embryonic lethality [10]. A similar approach proposes scanning the cumulative population-wide SNP genotyping data from genomic selection programs to identify haplotype regions indicative of a depletion in homozygosity [11]. The analysis includes the statistical evaluation of segregating haplotypes regarding the Hardy–Weinberg equilibrium (HWE) [11]. Such genomic regions harboring deficient homozygous haplotypes indicate potential causal variants for embryonic death or unwanted phenotypes in newborns, leading to the exclusion of homozygous animals from breeding programs [11]. This SNP-based reverse genetics approach has been used successfully in diverse breeds and species (e.g., [11,12,13,14,15,16,17,18,19,20,21,22,23,24,25,26,27,28,29,30,31]). Haplotypes never observed in homozygous state indicate the presence of recessive, predominantly embryonic lethal variants. Potential causative variants were detected by analyzing whole-genome or whole-exome sequencing data linked to the identified haplotypes (e.g., [10,19,20,21,22,23,24,25,26,27,28,29,30,32,33,34,35]). In general, these approaches are especially interesting for small populations where phenotypic information is sparse.

In Simmental-derived breeds in Central Europe, eight causal protein-changing variants for recessively inherited disorders are known so far. In German Fleckvieh cattle, a SNP-based reverse genetic study identified four deficient homozygous haplotypes located on chromosomes 1, 10, and 12 and proposed two candidate causal variants in two genes: the FH2-related frameshift variant in *SLC2A2* and the FH4-related missense variant in *SUGT1* [21]. In addition, a fifth haplotype is described, affecting the calf survival due to a congenital heart failure and severe liver damage (https://www.lfl.bayern.de/itz/rind/122227/index.php; accessed on 24 October 2021). Based on forward genetic studies, where affected individuals were examined, several causal variants for Mendelian disorders were found. A frameshift variant in *GON4L* was associated with the autosomal recessively inherited disorder dwarfism (OMIA 001985-9913) [36]. Furthermore, a nonsense variant in *PLD4* causing a recessive genodermatosis observed in German Fleckvieh (OMIA 001935-9913) and a missense variant in *OPA3* associated with a form of dilated cardiomyopathy predominantly affecting Swiss Fleckvieh (OMIA 000162-9913) were described [37,38]. In German Fleckvieh, a *MOCS1*-related form of arachnomelia was identified to be due to a frameshift variant (OMIA 001541-9913) [39]. Furthermore, the BH2-related *TUBD1* missense variant, known to cause juvenile mortality in Braunvieh cattle (OMIA 001939-9913), was also observed in German and Austrian Fleckvieh [23]. To the best of our knowledge, so far, no carriers for any of these eight deleterious alleles have been found in the purebred Swiss Simmental population.

In purebred Swiss Simmental cattle, no genomic analysis has yet been carried out to systematically identify recessively inherited harmful variants that affect female reproduction or calf rearing. Based on a reverse genetic screen, the present study aimed at a comprehensive analysis of available SNP and WGS data to identify genomic regions containing deficient homozygous haplotypes as well as linked candidate causal variants for hidden phenotypes.

## 2. Materials and Methods

The national breeding association provided us with the available SNP data, including all genotyped purebred Swiss Simmental cattle born after 2009 and their ancestors. The genomic positions of the markers relate to the latest cattle reference sequence ARS-UCD1.2 [40,41]. Due to the application of several routinely available SNP arrays ranging from 9000 to 150,000 SNPs, the data had to be imputed. Therefore, the software Fimpute v2.2 [42] was used with default parameters to increase the number of markers and correct for wrongly called markers. In order to assure the quality, SNPs with a minor allele frequency <0.01 were excluded from the dataset. Furthermore, SNPs that could not pass the quality measures for the call rate per SNP >0.99 were excluded and animals with call rates <0.8 were excluded too. Quality control was applied before and after imputation. This resulted in a final SNP data set of 114,890 markers for 9965 animals.

As a first step, haplotypes showing a deviation from the HWE, indicated by depletion of homozygosity, were identified. The first subset of data analyzed included only fully genotyped trios where the complete trio (sire, dam, and offspring) were genotyped (*n* = 2626), further called “trio” approach. The second dataset analyzed included genotyped trios where an offspring and two paternal animals (sire and maternal grandfather) were genotyped (*n* = 3969), subsequently called parent–grandparent “pgp” approach. Both data sets, trio and pgp, were used in screening for window size of 50 markers within the software snp1101 [43]. The analyzed haplotypes overlap, as the windows were continuously moved marker by marker. The snp1101 software used the Fisher exact test of HWE [44] to analyze the resulting haplotypes. Furthermore, the p-values were corrected for a false discovery rate with the Benjamini–Yekultieli method and a significance level of 5% was applied [45]. For each significant haplotype region, we selected the most significant haplotype (lowest p-value). Regarding the identification of candidate variants, haplotype regions were defined by the selected haplotypes and increased on both sides by 2 Mb in order to make sure that the regions of overlapping significant haplotypes were included.

Within the 11 regions detected, between 14 and 262 genes were annotated, giving a total of 669 genes. To these genes, information regarding associated phenotypes were collected from the various online databases: HUGO Gene Nomenclature Committee (https://www.genenames.org; accessed on 7 October 2021), Mouse Genome Informatics (http://www.informatics.jax.org; accessed on 7 October 2021), International Mouse Phenotyping Consortium (https://www.mousephenotype.org; accessed on 7 October 2021), Online Mendelian Inheritance in Man (https://www.omim.org; accessed on 7 October 2021), Online Mendelian Inheritance in Animals (https://omia.org/home/; accessed on 7 October 2021), and Decipher (https://www.deciphergenomics.org; accessed on 7 October 2021).

With the selected haplotypes, we predicted individual diplotypes that represent if an animal carries one, two, or no copies of the haplotype. Based on these diplotypes, we selected three carrier animals for whole-genome sequencing (WGS) for each haplotype region. Therefore, 23 Simmental cattle (1 female and 22 male) carrying one or more significant haplotypes were selected for this study. WGS data were prepared as previously described [46]. However, recalibration was performed with the variant catalogue of the 1000 Bull Genomes Project run 7 (BQSR file version 2) [47,48]. The 23 genomes sequenced in this study were submitted to the 1000 Bull Genomes project [47,48] and are therefore part of the 5116 animals in the recent variant catalogue (run9). This international dataset was used to evaluate candidate causative variants in a larger cohort and across breeds, to evaluate breed specificity as well as reduced homozygosity. Furthermore, we had access to an additional 115 publicly available genomes from the Swiss Comparative Bovine Resequencing project, deposited in the European Nucleotide Archive under project accession PRJEB18113, that were yet not added to the 1000 Bull Genomes project. The combined WGS dataset of 5231 genomes includes 62 purebred Swiss Simmental cattle.

In order to identify haplotype-associated candidate causal variants, linkage disequilibrium (LD) analysis was performed using plink v1.9 [49]. LD (r^2^) was calculated between the diplotype state and the WGS-derived genotypes for the 23 animals that were selected for WGS. Analysis was restricted to protein-changing variants, including variants annotated by SnpEff (version 4.3; [50]) with the following sequence ontology terms: missense_variant, start_lost, stop_gained (nonsense), stop_lost, stop_retained_variant, splice_acceptor_variant, splice_donor_variant, conservative_inframe_deletion, conservative_inframe_insertion, disruptive_inframe_deletion, disruptive_inframe_insertion, exon_loss_variant, frameshift_variant, and gene_fusion.

Furthermore, to improve the understanding of candidate causal variants, their base conservation scores from UCSC database called PhyloP and PhastCons were applied [51,52]. To apply these values, firstly, the variants of the bovine genome needed to be mapped to the human genome 38 [53] with the tool LiftOver of the UCSC tools. Secondly, the conservation scores of 99 vertebrates for these human positions were obtained. Additionally, the effects of protein-changing variant were estimated using PROVEAN [54] and PredictSNP [55].

## 3. Results

In this reverse genetic study, we used SNP data to identify haplotypes showing a depletion in homozygosity and applied WGS data to pinpoint candidate causal variants in purebred Swiss Simmental animals. Reduced homozygosity due to hidden recessive variants in cattle could, so far, only be causally explained by coding variants. Therefore, we focused on variants having a moderate impact, such as missense variants, conservative in-frame insertions and deletions up to a size of 50 bp, as well as on all other protein-changing with high impact including loss-of-function variants, such as stop-gains (nonsense), splice site-disrupting SNVs, frameshift indels in a coding sequence, or deletions that remove coding exons.

### 3.1. Identification of Deficient Homozygous Haplotypes in Swiss Simmental Cattle

**Figure 1:**
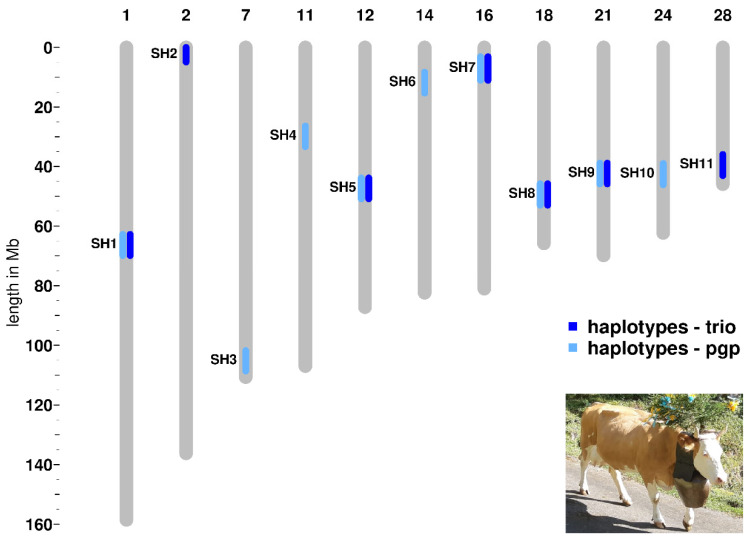
Genomic co-localization of the deficient homozygous haplotype regions detected in Swiss Simmental cattle using two different approaches. A typical breed-specific cow is shown in the lower right corner.

We detected seven haplotype regions with the trio approach and nine haplotype regions applying the pgp approach (Table 1, Figure 1, Table S1). Five of these haplotype regions appear in both analyses. We named the haplotype regions, in accordance to previous studies performed in other cattle breeds, as Simmental Haplotypes (SH) 1 to 11 [22] (Table 1, Table S1). All 11 identified haplotypes show a deficiency of at least 85 percent of the expected homozygous animals within the studied Swiss Simmental population (Table 1, Table S1). Four selected haplotypes (SH5, SH7, SH8, and SH10) presented a complete deficit of observed homozygous animals, whereas the others showed a partial deficiency ranging from 85 to 96% of the expected homozygotes (Table 1; Table S1). The average length of the eleven haplotypes is 1.17 Mb and ranges from 0.73 to 1.94 Mb (Table 1).

### 3.2. Identification of Candidate Genes in Haplotype Regions

The intensive analysis of all annotated protein-coding genes in the defined haplotype regions, extended by 2 Mb on each side, led to a comprehensive list of candidate genes possibly affecting either prenatal or postnatal lethality or associated sub-lethal phenotypes. We extracted 145 positional candidate genes of special interest, as they are associated with mammalian autosomal recessive disorders in human, mice, or other animals and listed in the consulted databases (Table S2). Loss-of-function mouse models of many of these genes have revealed defects that affect embryonic or perinatal to pre-weaning survival and therefore represent suitable functional candidates for this study. The presented short list of the 43 most probable candidate genes includes all genes that are associated with sub-lethal or lethal phenotypes (Table 2).

### 3.3. Identification of Candidate Causal Variants

For three deficient homozygous haplotypes (SH5, SH8, and SH9), by linkage disequilibrium analysis, we found perfectly linked (r^2^ = 1) candidate causal variants. These three haplotypes were detected with both the pgp and the trio approach. For each of these haplotypes, we propose a protein changing SNV (Table 3; Table S3). These three variants never occur in homozygous state in the analyzed 5231 bovine genomes of various cattle breeds (Table S3). Interestingly, the SH8-associated variant is apparently specific for Swiss Simmental; however, the variants associated with SH5 and SH9 occur sporadically in single animals of some other breeds (Table S3).

**Figure 2:**
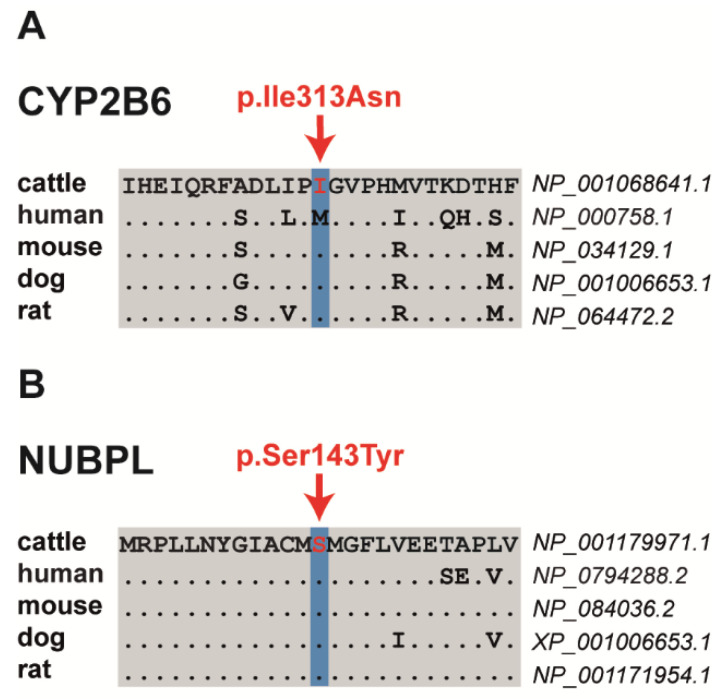
Multiple sequence alignment of the amino acid sequences of CYP2B6 (**A**) and NUBPL (**B**) around the position of the missense variants (shown in red), indicating evolutionary conservation across all species.

Among the three proposed non-synonymous variants are two missense variants altering evolutionary conserved residues and a frameshift variant that significantly truncates the encoded protein. The SH8-related SNV in exon 4 of the bovine *cytochrome P450 family 2 subfamily B member 6* (*CYP2B6*) gene on chromosome 18 at position 50296371 is a missense variant (NM_001075173.1: p.Ile313Asn) that was predicted by PROVEAN to have a deleterious effect (Table 3, Table S3). The SH9-related SNV located in exon 6 of the bovine *nucleotide binding protein-like* (*NUBPL*) gene on chromosome 21 at position 42154344 represents a missense variant (NM_001193042.1: p.Ser143Tyr) predicted to be deleterious by PROVEAN and PredictSNP (Figure 2, Table 3, Table S3). Both presented missense mutations altering evolutionary conserved amino acids (Figure 2). The SH5-associated 1 bp insertion located in exon 16 of the bovine *DIS3 homolog, exosome endoribonuclease and 3’-5’ exoribonuclease* (*DIS3*) gene on chromosome 12 at position 47511687 represents a loss-of-function variant. It was predicted to result in a frameshift after isoleucine 678 with a premature stop codon (NP_025000110.1: p.Ile678AsnTer2), resulting in a significantly shortened amino acid sequence, if expressed, when compared with the wild-type protein (Table 3; Table S3). In addition, the comparative DNA sequence approach (PhyloP and phastCons) showed a high conservation across species for all three variant positions (Table S3).

## 4. Discussion

For the first time, the genomic data of the current Swiss Simmental dual-purpose cattle population were analyzed for reduced homozygosity due to hidden recessive monogenic variants and validated using the international variant catalogue of the 1000 Bull Genomes Project. In addition to environmental factors, inherited deleterious variants lead to natural or artificial selection against homozygous individuals, which also explains embryonic lethality, reduce rearing success, or the exclusion from the breeding population due to poor development. Unfortunately, these phenomena are not systematically monitored and are therefore difficult phenotypes to study. Although reduced reproductive success can theoretically be detected in the sires estimated breeding values for certain fertility traits, these effects are only noticeable for deleterious alleles that have reached a high frequency in the population [56]. To overcome this issue, we performed a genome-wide missing homozygosity scan, revealing eleven haplotype regions with considerable homozygous depletion. After subsequent mining of genome sequence data for candidate causal variants, we propose three non-synonymous variants that probably cause the obvious deficiency of homozygous animals.

We applied two different approaches, a trio and pgp approach, that include genotyped trios and applies a Fisher exact test. Therefore, we were able to detect haplotypes that segregate at a lower allele frequency. As expected, we found more haplotype regions with the pgp approach in comparison to the trio approach, most likely because more genotyped groups were available. Nevertheless, the trio approach, detecting two regions not found with pgp, appears very powerful, probably because it directly traces the inheritance of the haplotypes. Previous approaches used the assumption of random mating and the deviation from HWE based on allele frequencies or used the deviation from expected number of homozygous offspring based on the haplotype state of the sire and maternal grandsire [14,20]. In contrast, the herein applied trio approach allows performing such an analysis with a small population such as Simmental. The five haplotype regions that were found in both analyses are the most probable genome regions harboring hidden harmful variants. Especially the haplotypes that never occur in homozygous state led to the suspicion of embryonic lethal variants segregating in the population. It is suspected that some haplotypes arose due to imputation errors introduced due to genotyping bias, SNP density/panel, sample size, and a bias introduced by the chosen software [57]. This would explain the haplotype regions SH3 and SH11 that arose in regions for which we could not identify any plausible functional candidate genes. Otherwise, we were able to identify candidate genes within all missing homozygous regions. Unfortunately, effect estimations of the haplotypes towards traits of female fertility and rearing success were not reasonable, as the available phenotypic data is currently limited. To avoid the detection of mostly sporadic associations rather than actual effects, it is planned to conduct such haplotype association studies in the future.

Candidate causal variants are proposed for the haplotypes SH5, SH8, and SH9 in the genes *DIS3*, *CYP2B6,* and *NUBPL*, respectively. These variants all show complete depletion in homozygosity, perfect LD to the associated haplotype, and high conservation scores when compared across 99 genomes, indicating their importance in basic biological functions. As in other mammals, it is expected that ~100 harmful recessive variants will be found per individual in cattle, of which up to five of these impact essential genes and cause embryonic lethality or severe disease when homozygous [10]. Nevertheless, it is recognized that it is very difficult to clarify the actual deleterious functions of these variants, although given the genes involved, it is assumed that these variants influence fitness. Reverse genetic screens to identify genes with major effects, as used in the current study, are therefore helpful to assign function to variants in candidate genes and/or so far less characterized genes such as *DIS3* and *CYP2B6*.

The SH5-related loss-of-function variant found in bovine *DIS3,* most likely leading to missing homozygosity, represents the first time a pathogenic variant that most likely causes embryonic lethality has been identified. If the mutant mRNA transcript were to escape nonsense-mediated decay, even if this truncated protein was expressed, it would lack roughly 30 percent of the C-terminal part, and therefore it is not expected to contribute any function. *DIS3*, also known as ribosomal RNA-processing protein 44 (RRP44), is a RNase II/R-like enzyme located primarily in the nucleus (Table S3) [58]. The protein has catalytic function in the RNA exosome complex, which is responsible for 3′-end processing and RNA degradation of a broad variety of RNAs [58,59]. Biological functions are associated with RNA metabolism, mitotic control, spindle-fiber formation, antibody diversification, microtubule production, and growth and development [60]. Recently, pathogenic variants in genes encoding both structural and catalytic subunits of the RNA exosome have been linked to human disease, such as *EXOSC3* and *EXOSC8* related forms of pontocerebellar hypoplasia, representing recessive neurodegenerative diseases [61]. To our knowledge, the *DIS3* gene has not yet been associated with Mendelian diseases, but variants are reported to be associated with various types of cancers and multiple myeloma [60,61,62]. Interestingly, the book of Fasken et al. (2020) provides a summary of the most common variants in *DIS3* which all occur in heterozygous state, are associated with multiple myeloma, and seem to have mild effects only, while Tomecki et al. (2014) suggests the potential lethality of mutations in the PIN domain of *DIS3*. In *Drosophila,* a knock-down model led to wingless animals implicating an important role in development [63]. Nevertheless, the inactivation of *DIS3* in B cells was shown to lead to an increase in unbalanced DNA translocations [64]. Lastly, public databases for mouse phenotypes indicate a complete pre-weaning lethality of *DIS3* knock-out mice (https://www.mousephenotype.org/data/genes/MGI:1919912; http://www.informatics.jax.org/marker/MGI:1919912; accessed on 7 October 2021).

Despite a long list of candidate genes located within the SH8-region, we propose a missense variant in the *CYP2B6* gene as a candidate causal variant. The main reason for this is the perfect LD to the haplotype, the complete absence of homozygous animals, and the prediction of the DNA position to be highly conserved and the amino acid exchange to be deleterious. Regarding the gene function, CYP2B6 is a protein of the cytochromes P450 subfamily 2B (HGNC: 20604). This enzyme is known to be of importance for drug metabolism, as well as endogenous compounds, environmental toxins, and other substances [65,66]. For example, the susceptibility to Efavirenz depends on the individual *CYP2B6* genotype (OMIM: 123930). Several SNV were detected in human that are associated with the expression level and activity (increased and decreased) of *CYP2B6* with a population-wide importance [65] (https://www.pharmvar.org/gene/CYP2B6; accessed on 26 October 2021). In monkeys and human, it was shown that *CYP2B6* is expressed in the brain and affected by nicotine and alcohol consumption; however, its neurological function remains unclear [65,66,67]. The protein is also expressed in the placenta [68] and it was shown that the pregnancy hormone estradiol induces the expression of *CYP2B6* [69]. Nevertheless, what the function and importance of *CYP2B6* is in maintaining pregnancy is unclear. As the herein identified bovine variant is predicted to be deleterious, we speculate that function of CYP2B6 might be impaired during development.

Lastly, we propose the missense variant in the bovine *NUBPL* gene, exchanging a strongly conserved residue predicted to be deleterious and affecting a highly conserved nucleotide, to be causal for the deficit of homozygosity of SH9. As we observe few haplotype carriers in our data, we speculate that the effect of the variant is not fully penetrant or that signs of poor development appear later in life, after initial genotyping of young animals. NUBPL, also known as *iron-sulfur protein required for NADH dehydrogenase* (*IND1*), is a protein that is vital for the assembly of the respiratory complex I [70]. More precisely, the NUBPL supplies Fe/S clusters to the respiratory complex I and thereby ensures that important subunits are delivered to build the whole complex [70]. In human, pathogenic variants affecting *NUBPL* are associated with the autosomal recessive mitochondrial complex I deficiency disorder (OMIM: 613621, 618242) [71,72,73]. These variants include missense, frameshift and splice site variants, as well as small and large insertions and deletions. Clinical symptoms of mitochondrial complex I deficiency include, among others, ataxia, dysarthria, hypotonia, nystagmus, spasticity, and tremor [73]. Furthermore, variants in *NUBPL* were hypothesized as risk factors for Parkinson’s disease [74]. A mouse model identified the necessity of the protein as knock-out alleles led to homozygous lethality (MGI: 1924076) [75].

However, if the proposed variants are indeed depleted in a number of homozygous animals, it needs to be evaluated by further genotyping of larger cohorts before implementation into selection schemes. This is planned by adding the variants to a custom array for genotyping larger cohorts of further animals, both Swiss Simmental as well as other local cattle populations. An alternative approach to confirm the absence of homozygous animals, particularly in the offspring of carrier-by-carrier mating’s, would strongly support the deleterious nature of the variants as shown before [10]. In particular, for the *NUBPL*-associated variant, the observed homozygous animals should be examined in detail.

The chosen approach requires that the haplotype and the causative variant are in near perfect linkage disequilibrium and, obviously, this is not always the case. This could be an explanation for the fact that we could not identify any potentially causal variants in the other haplotype regions. An alternative approach, therefore, is to mine the genome sequence data for candidate variants, e.g., loss-of-function in the listed essential candidate genes, and to genotype these directly in large cohorts of Swiss Simmental cattle. Using this approach, nine causal variants were uncovered in cattle that would not have been detected using SNP-based haplotype approaches [10].

Finally, another drawback of our study was the restriction to protein-changing variants. Moreover, we had no evidence for perfectly linked non-coding regulatory variants, as well as the limitation to consider only SNV and short indels, overlooking possible larger structural variants.

## 5. Conclusions

In the presented project, we mined SNP and WGS data by applying a reverse genetic approach. Without any phenotypic evidence but mining the data of almost 10 thousand SNP genotyped Swiss Simmental cattle and more than 5200 WGS animals from a variety of breeds, we propose three candidate variants in the genes *DIS3*, *CYP2B6,* and *NUBPL* causing embryonic lethality and/or yet unknown recessive developmental disorders. After phenotypic validation of these variants, selection against these variants is recommended.

## Figures and Tables

**Table 1:** List of deficient homozygous haplotypes in Swiss Simmental cattle.

					Number of Homozygotes			
Haplotype ^a^	Analysis	Chr	Position (Mb) ^b^	Length (Mb)	Observed	Expected	Deficiency (%)	Allele Frequency
SH1	trio and pgp	1	65.793–66.891	1.10	3	17	85	0.042
SH2	trio	2	1.191–1.920	0.73	1	16	94	0.040
SH3	pgp	7	104.784–105.640	0.86	1	17	94	0.041
SH4	pgp	11	29.389–30.353	0.96	4	22	85	0.047
SH5	trio	12	46.828–47.837	1.01	0	38	100	0.062
	pgp		46.831–47.843	1.01				
SH6	pgp	14	11.304–12.373	1.07	2	15	88	0.039
SH7	trio	16	6.120–8.011	1.89	0	30	100	0.055
	pgp		6.122–8.060	1.94	5	111	96	0.106
SH8	pgp	18	48.763–50.005	1.24	0	11	100	0.0337
	trio		48.806–50.017	1.21				0.0338
SH9	trio and pgp	21	41.855–42.851	1.00	2	19	96	0.043
SH10	pgp	24	41.945–43.140	1.20	0	10	100	0.032
SH11	trio	28	38.937–40.069	1.13	1	12	92	0.035

^a^ SH meaning Simmental Haplotype. ^b^ in accordance with the reference sequence ARS-UCD1.2.

**Table 2:** List of candidate genes known to cause recessive disorders with lethal and sub-lethal phenotypes in human.

Haplotype	Gene	Gene Description	Recessive Disorder
SH1	*IQCB1* ^§^	IQ motif containing B1	Senior-Loken syndrome 5
	*MYLK* ^§^	myosin light chain kinase	Megacystis-microcolon-intestinal hypoperistalsis syndrome 1
	*CASR* *	calcium sensing receptor	Hyperparathyroidism, neonatal
	*POGLUT1* *	protein O-glucosyltransferase 1	Limb-girdle muscular dystrophy-dystroglycanopathy
	*TIMMDC1* *	translocase of inner mitochondrial membrane domain containing 1	Mitochondrial complex I deficiency, nuclear type 31
SH2	*HERC2* *^,§^	HECT and RLD domain containing E3 ubiquitin protein ligase 2	Mental retardation, MRT 38
	*OCA2* *^,§^	OCA2 melanosomal transmembrane protein	Albinism, brown oculocutaneous
SH4	*CRIPT* *	CXXC repeat containing interactor of PDZ3 domain	Short stature with microcephaly and distinctive facies
	*EPCAM* *	epithelial cell adhesion molecule	Congenital diarrhea with tufting enteropathy
	*FSHR* *^,§^	follicle stimulating hormone receptor	Ovarian dysgenesis 1
	*MSH2* ^§^	mutS homolog 2	Mismatch repair cancer syndrome 2
	*MSH6*	mutS homolog 6	Mismatch repair cancer syndrome 3
	*PIGF* *	phosphatidylinositol glycan anchor biosynthesis class F	Onychodystrophy, osteodystrophy, impaired intellectual development, and seizures syndrome
	*PPP1R21* *	protein phosphatase 1 regulatory subunit 21	Neurodevelopmental disorder with hypotonia, facial dysmorphism, and brain abnormalities
	*TTC7A* *	tetratricopeptide repeat domain 7A	Gastrointestinal defects and immunodeficiency syndrome
SH5	*PIBF1* *	progesterone immunomodulatory binding factor 1	Joubert syndrome 33
SH6	*MYC* *	MYC proto-oncogene, bHLH transcription factor	Burkitt lymphoma, somatic
SH7	*CFH*	complement factor H	Basal laminar drusen, complement factor H deficiency
	*CR2* ^§^	complement C3d receptor 2	Immunodeficiency, CVID7
	*IL10* ^§^	interleukin 10	Critical role in the control of immune responses
SH8	*ARHGEF1* ^§^	Rho guanine nucleotide exchange factor 1	Immunodeficiency 62
	*BCKDHA* *	branched chain keto acid dehydrogenase E1 subunit alpha	Maple syrup urine disease, type Ia ^@^
	*B9D2* *	B9 domain containing 2	Meckel syndrome 10, Joubert syndrome 34
	*COQ8B* *	coenzyme Q8B	Nephrotic syndrome, type 9
	*DLL3* ^§^	delta-like canonical Notch ligand 3	Spondylocostal dysostosis 1
	*ERF* *	ETS2 repressor factor	Spondylocostal dysostosis 1
	*ETHE1* ^§^	ETHE1 persulfide dioxygenase	Ethylmalonic encephalopathy
	*LTBP4* ^§^	latent transforming growth factor beta binding protein 4	Cutis laxa, autosomal recessive, type IC
	*MEGF8* *	multiple EGF-like domains 8	Carpenter syndrome 2
	*PLEKHG2*	pleckstrin homology and RhoGEF domain containing G2	Leukodystrophy and acquired microcephaly
	*RYR1* *	ryanodine receptor 1	Neuromuscular disease
	*SMG9* *	SMG9 nonsense mediated mRNA decay factor	Heart and brain malformation syndrome
	*SPINT2* *	serine peptidase inhibitor, Kunitz type 2	Diarrhea 3, secretory sodium, congenital, syndromic
	*SPTBN4*	spectrin beta, non-erythrocytic 4	Neurodevelopmental disorder with hypotonia, neuropathy, and deafness
	*TGFB1* *	transforming growth factor beta 1	Inflammatory bowel disease, immunodeficiency, and encephalopathy
	*TIMM50* *	translocase of inner mitochondrial membrane 50	3-methylglutaconic aciduria, type IX
	*XRCC1* *	X-ray repair cross complementing 1	Spinocerebellar ataxia, SCAR26
SH9	*NUBPL* *	nucleotide binding protein-like	Mitochondrial complex I deficiency, nuclear type 21
SH10	*AFG3L2* *	AFG3-like matrix AAA peptidase subunit 2	Spastic ataxia 5
	*LAMA1* *	laminin subunit alpha 1	Poretti–Boltshauser syndrome
	*MC2R* ^§^	melanocortin 2 receptor	Glucocorticoid deficiency due to ACTH unresponsiveness
	*NDUFV2* *	NADH:ubiquinone oxidoreductase core subunit V2	Mitochondrial complex I deficiency, nuclear type 7
	*PIEZO2* *	piezo type mechanosensitive ion channel component 2	Arthrogryposis, distal, with impaired proprioception and touch

* Homozygous lethal with complete penetrance, ^§^ homozygous lethal with incomplete penetrance, ^@^ disorder described in cattle (OMIA-ID: 000627-9913).

**Table 3:** Candidate causal variants for deficiency of homozygotes in Swiss Simmental cattle.

Haplotype	Gene	OMIM	Variant	Transcript ^a^	Coding DNA Change	Predicted Protein Change
SH5	*DIS3*	607533	1 bp insertion (frameshift)	XM_025000110.1	c.2032dupA	p.Ile678AsnTer2
SH8	*CYP2B6*	123930	SNV (missense)	NM_001075173.1	c.938T > A	p.Ile313Asn
SH9	*NUBPL*	613621	SNV (missense)	NM_001193042.1	c.428C > A	p.Ser143Tyr

^a^ According to the NCBI Annotation Release 106 (National Center for Biotechnology Information, 2018b).

## Data Availability

The SNP data of Swiss Simmental cattle are owned by the breeding association swissherdbook. Therefore, we ask interested people to contact the authors or the breeding association directly in order to gain access to the SNP data. The WGS data are publicly available at the European Nucleotide Archive under project accession PRJEB18113.

## Supplementary Materials

The following are available online at https://www.mdpi.com/article/10.3390/ani11123535/s1, Table S1: Haplotype information of SH1 to SH11, Table S2: Comprehensive list of candidate genes located in the haplotype regions, including gene information from MGI, IMPC, OMIM, and OMIA, Table S3: Comprehensive list of potential candidate causal variants.
